# Effects of puerarin on chronic inflammation: Focus on the heart, brain, and arteries

**DOI:** 10.1002/agm2.12189

**Published:** 2021-12-15

**Authors:** Li Zhang, Lisheng Liu, Mingyi Wang

**Affiliations:** ^1^ Department of Cardiology The First Affiliated Hospital of Guangdong Pharmaceutical University Guangzhou China; ^2^ National Centre for Cardiovascular Disease The Beijing Hypertension League Institute Beijing China; ^3^ Laboratory of Cardiovascular Science Intramural Research Program National Institute on Aging National Institutes of Health BRC Baltimore Maryland USA

**Keywords:** artery, brain, heart, inflammation, puerarin

## Abstract

Age‐associated increases in physical and mental stress, known as allostatic load, could lead to a chronic low‐grade inflammation in the heart, brain, and arteries. This low‐grade inflammation potentially contributes to adverse structural and functional remodeling, such as intimal medial thickening, endothelial dysfunction, arterial stiffening, cardiac hypertrophy and ischemia, and cognitive decline. These cellular and tissue remodeling is the fertile soil for the development of age‐associated structural and functional disorders in the cardiovascular and cerebrovascular systems in the pathogenesis of obesity, type II diabetes, hypertension, atherosclerosis, heart dysfunction, and cognitive decline. Growing evidence indicates that puerarin, a polyphenol, extracted from *Puerara Labota*, efficiently alleviates the initiation and progression of obesity, type II diabetes, hypertension, atherosclerosis, cardiac ischemia, cardiac arrythmia, cardiac hypertrophy, ischemic stroke, and cognition decline via suppression of oxidative stress and inflammation. This mini review focuses on recent advances in the effects of puerarin on the oxidative and inflammatory molecular, cellular, tissue events in the heart, brain, and arteries.

## Graphical abstract

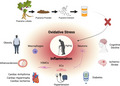

## 1 INTRODUCTION

**FIGURE 1:**
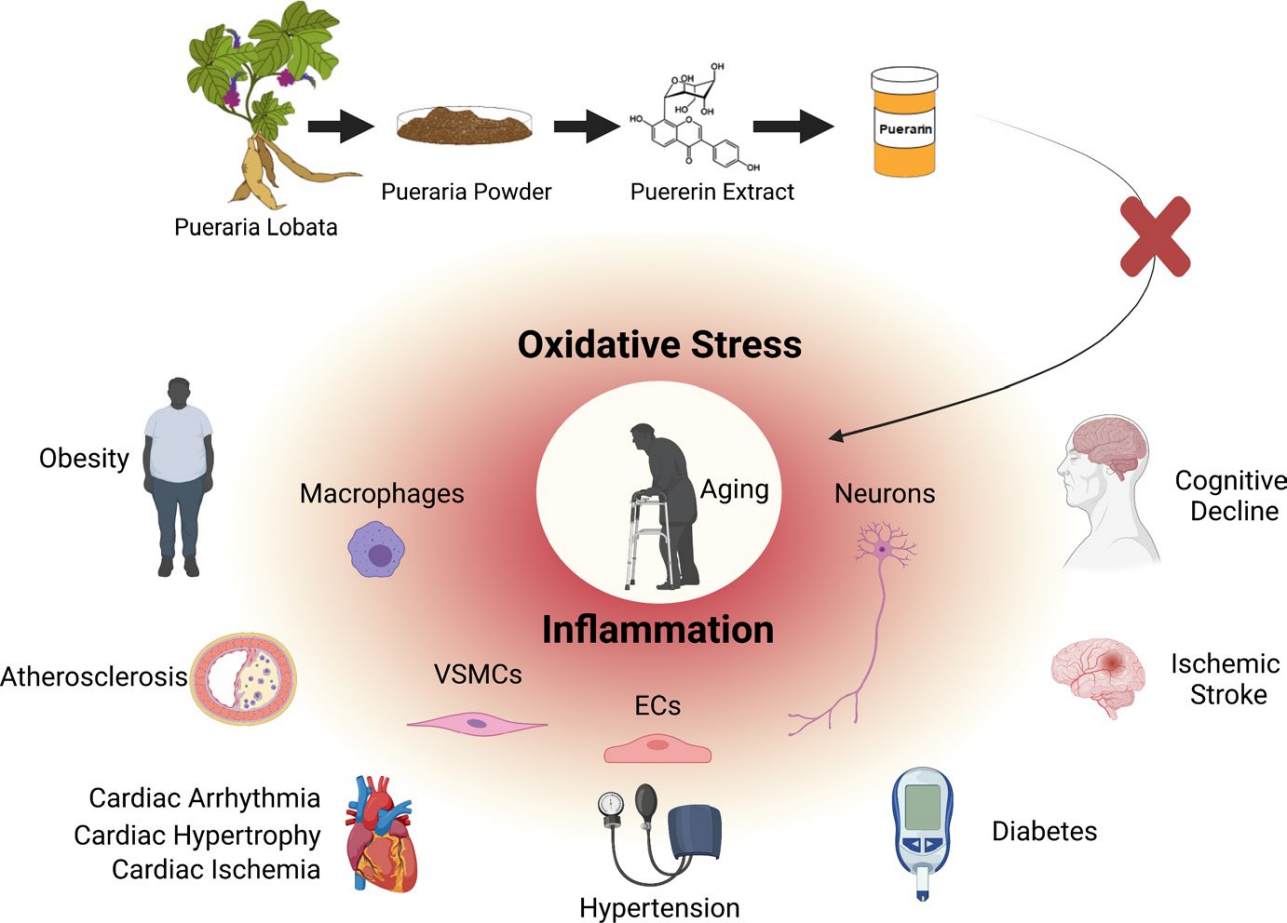
Illustration of puerarin alleviation of oxidative stress and adverse inflammatory cell and tissue events in the heart, brain, and arteries in the pathogenesis of obesity, diabetes, hypertension, atherosclerosis, heart disorders and cognitive decline, which are significantly increased with advancing age. Note: This illustration was generated using BioRender program (https://biorender.com/) by Kimberly Raginski McGraw of Laboratory of Cardiovascular Science, National Institute on Aging, National Institutes of Health

Aging is a chronic low‐level proinflammatory process in response to the physical and mental stress, known as allostatic load, that results in the progressive functional decline and structural degeneration of cells, tissues, and organs, such as in the heart, brain, and arteries.^1^, ^2^, ^3^, ^4^, ^5^ These structural and functional alterations are the fertile soil for developing age‐associated diseases, such as hypertension, atherosclerosis, heart failure, and cognitive decline.^1^, ^2^, ^3^, ^4^, ^6^ Growing evidence indicates that it is impossible to stop chorological aging, per se, but slowing down the rate of biological aging is entirely achievable. Traditional Chinese medicine, characterized by nourishing life, which has its role in anti‐aging and age‐related disorder, is being recognized by the modern scientific and medical communities.^7^ Puerarin, is an isoflavonoid extract, from traditional Chinese medicine *Puerarae Lobatae*.^8^ Growing evidence reveals that an administration of puerarin efficiently fights age‐related cellular and molecular inflammatory events in the cardiovascular and cerebrovascular systems (Figure 1).^7^, ^8^, ^9^, ^10^ Puerarin further diminishes the structural and functional adverse remodeling in the cardiovascular system, which can eventually fights diabetes, hypertension, atherosclerosis, and stroke, which are markedly increased with advancing age (Figure 1). ^2^, ^10^, ^11^ This mini review addresses recent advances in how puerarin combats the structural and functional inflammatory remodeling in the age‐associated cardiovascular and cerebrovascular disorders.

## 2 PUERARIN ALLEVIATES THE CLINICAL RELEVANCE OF CHRONIC INFLAMMATION

### 2.1 Obesity, metabolic syndrome, and diabetes mellitus

Obesity, metabolic syndrome, and diabetes mellitus are major risk factors for the initiation and progression of cardiovascular and cerebrovascular diseases and are dramatically increased with advancing age in modern society.^12^ In obese mice treated with puerarin for 8 months, circulating levels of isoflavone metabolites were significantly increased; fasting blood glucose levels were markedly reduced; and glucose intolerance and insulin resistance were pronouncedly improved.^13^ In addition, in rats fed a high fat diet and treated with puerarin for 8 weeks, body weight was remained stable, glucose‐insulin intolerances were significantly improved, resistin abundance was markedly reduced, and adipokine secretion was greatly improved.^14^ Notably, increased resistin has been reported to be involved in insulin resistance; adipokines, such as leptin, regulates energy balance by inhibiting hunger and subsequently prevent an increase in body weight.^15^, ^16^ These findings suggest that puerarin is a beneficial adjuvant medicine for prevention and treatment of obesity and obesity associated type II diabetes.

Indeed, in rats fed a combination of a high fat diet and streptozocin (STZ), an animal model of type II diabetes, puerarin treatment significantly decreased hepatic glucose levels, maintained lipid homeostasis, reduced fasting blood glucose and hemoglobulin A1C (HA1C) levels, and inhibited hepatic gluconeogenesis.^17^, ^18^ These beneficial effects are closely associated with the activation of the phosphoinositide 3‐kinase (PI3K)/AKT (protein kinase B) pathway.^17^, ^18^ In addition, puerarin significantly reduced the cytotoxicity of reactive oxygen species (ROS) via an enhancement of catalase super oxide mutase (SOD), prevented the development of the apoptosis of pancreatic β‐cells, increased the survival capacity of β‐cells, and promoted the secretion of insulin from pancreatic islets, finally contributing to the decreases in body weight and blood glucose levels.^19^, ^20^, ^21^ Furthermore, puerarin blocked the preadipocyte differentiation and the glucose uptake of adipocytes in insulin resistant conditions.^22^ Interestingly, puerarin reduced the apoptosis of endothelial cells (ECs) induced by tissue necrosis factor alpha 1 (TNF‐α1), which were associated with increase of peroxisome proliferator‐activated receptor gamma (PPAR‐γ) expression.^22^ It is known that TNF‐α1 and PPAR‐γ directly or indirectly reciprocally affect the occurrence and development of insulin resistance by modulating glucose metabolism, insulin signaling transduction, and chronic inflammatory responses.^22^, ^23^, ^24^, ^25^ In addition, puerarin retarded the TNF‐α1‐induced accumulation of intracellular calcium in endothelial cells (ECs), which likely causes cellular necrosis.^22^ These findings suggest that the administration of puerarin could improve type II diabetes‐related cellular inflammatory remodeling, including in β‐cells, adipocytes, and ECs.

Notably, both structural and functional disorders of the myocardial, renal, and skeletal muscle tissue are major complications for type II diabetes. Puerarin diminished hyperglycemia and exerted a cardiac protective role by reducing cardiomyocyte hypertrophy and cardiac fibrosis in diabetic rats induced by STZ.^26^, ^27^ Puerarin significantly reduced the expression of the receptor for advanced glycation end products (RAGE), improved autophagy function via upregulation of its essential components of beclin, microtubule‐associated protein 1A/1B‐light‐chain 3 (LC3II), and autophagy related 5 (ATG5), increased the antioxidant activity of heme oxygenase (HO) and the anti‐aging molecule SIRT1 expression, alleviated endoplasmic reticulum (ER) stress, reduced hyperglycemia, and eventually improved renal function in diabetic rats.^28^, ^29^, ^30^ In addition, puerarin lowered insulin resistance, and nuclear factor kappa B (NF‐κB) mediated inflammation, and prevented the incidence of gestational diabetes in rats fed a high fat diet.^31^ Taken together, the above studies indicate that puerarin has potential as a functional food therapeutic for the obesity, metabolic syndrome, diabetes mellitus, and their complications.

### 2.2 Hypertension

Hypertension is one of the leading causes of cardiovascular and cerebrovascular diseases; and is dramatically increased with advancing age that greatly affects health and wellbeing in the world.^12^ Hypertension doubles the risk of coronary heart disease, congestive heart failure, and ischemic stroke.^12^ Puerarin effectively lowered the levels of systolic blood pressure (SBP) and diastolic blood pressure (DBP) as well as heart rate (HR) in spontaneous hypertensive rats (SHRs).^32^ Furthermore, puerarin enhanced the expression of nitric oxide (NO), cyclic guanosine 3′,5′‐monophosphate (cGMP) levels, and phosphorylate endothelial nitric oxide synthase (p‐eNOS), whereas diminished vasoconstrictive angiotensin II (Ang II) type 1 receptor (AT1) abundance, contributing to vasodilation and improvement of endothelial function in the hypertensive rats.^32^ Puerarin also reduced the mRNA levels of the profibrogenic signaling molecule mothers against decapentaplegic homolog 3 (SMAD 3) while raised the mRNA levels of the anti‐fibrogenic signaling molecule SMAD 7, contributing to a decrease of myocardial fibrotic transforming growth factor‐beta1 (TGF‐β1) fibrogenic signaling and myocardial fibrosis in hypertensive animals.^33^

In hypertensive rats induced by aortic banding, puerarin administration lowered SBP while elevated the ratio of an apoptotic inhibitor B‐cell lymphoma protein 2 (Bcl‐2)/ a promoter Bcl‐2‐associated X (BAX), thus inhibiting the development of cardiomyocyte apoptosis and restoring myocardial muscle and myocardial function.^27^ Additionally, puerarin markedly reduced the expression of atrial natriuretic peptide (ANP) and brain natriuretic peptide (BNP) hypertrophic fetal genes, and subsequently diminished the development of cardiac hypertrophy via an inhibition of the PI3K/JNK signaling pathway in hypertensive rats.^27^

In hypertensive rats fed a high salt diet, puerarin treatment markedly improved acetylcholine mediated vasorelaxation through inhibiting the master inflammatory NF‐κB activation and inducing insulin‐sensing a serine/threonine protein kinase Akt and endothelial nitric synthase (eNOS) activation; and this treatment also prevented adverse cardiovascular remodeling, including cardiac hypertrophy, cardiac fibrosis, and arterial thickening.^26^ Puerarin cardiovascular beneficial effects were also associated with an inhibition of both aortic and cardiac p‐ERK1/2 MAP kinase signaling pathway.^26^

In hypertensive Sprague Dawley (SD) rats induced by Ang II infusion, puerarin treatment substantially prevented arterial intimal medial thickening and markedly improved endothelial dependent vasorelaxation, and greatly retarded the development of cardiac hypertrophy.^34^ Further study shows that at the molecular level, puerarin increased the eNOS activity and the production of NO, whereas decreased nicotinamide adenine dinucleotide phosphate (NADPH) oxidase via the suppression of its integral subunits gp91 and p22 phox, contributing to a decrease in the production of ROS; and inhibited the fibrogenic TGF‐β1 activation and the development of fibrosis in the myocardium.^34^ Thus, puerarin has the potential to prevent the adverse structural and functional remodeling of hypertensive arterial and cardiac tissue, such as arterial thickening, endothelial dysfunction, cardiac fibrosis, and cardiac hypertrophy.

### 2.3 Atherosclerosis

Atherosclerosis is a chronic, lipid‐driven, inflammatory disease of the arterial wall causing multifocal plaque development with or without thrombosis and is dramatically increased with aging.^5^ In an atherosclerotic rabbit model, puerarin treatment inhibited lipid deposition and NF‐kB inflammatory activation, eventually preventing the progression of atherosclerosis.^35^ In mice fed a high fat diet, puerarin treatment lowered serum lipid levels and body weight; reduced fatty acid synthase, adenosine monophosphate‐activated protein kinase (AMPK), and carnitine acyltransferase activation; and prevented lipid accumulation in the arterial wall.^23^ Furthermore, in atherosclerotic rabbits, puerarin administration significantly decreased total cholesterol, triglyceride, and low density lipid‐cholesterol levels, and subsequently reduced the expression of proliferating nuclear antigen (PCNA) and platelet derived growth factor (PDGF) in vascular smooth muscle cells (VSMC), and consequently inhibited the proliferation of VSMCs, contributing to a reduction of the burden of atherosclerotic plaques.^36^ Increases in either PDGF or PCNA drives the proliferation of VSMCs, which is key to the initiation and progression of atherosclerotic plaques.^36^, ^37^ In hypercholesteremic rats, puerarin treatment also reduces cholesterol levels while it increases the eNOS expression, contributing to the reduction of atherogenesis in the aortic wall.^38^ Activated eNOS facilitates the production of NO in ECs, which is a key molecular process for the antioxidant reaction in favor of the survival of ECs and integrity of the endothelium. The structural and functional integrity of the endothelial layer is a crucial cellular barrier that prevents circulating lipid infiltration into sub‐endothelial space and maintains arterial wall health.^32^, ^39^ Taken together, puerarin is a potential herbal medicine capable of curbing atherogenesis and atherosclerotic complications.

### 2.4 Myocardial ischemia and myocardial infarction

Myocardial ischemia is a condition in which there is an inadequate supply of blood and oxygen to a portion of the myocardium, and usually occurs when a balance between myocardial oxygen supply and demand is lost. The incidence of myocardial ischemia is greatly increased in the elderly.^2^ In a myocardial ischemic mice model, puerarin treatment improved myocardial infarction because this medicine effectively inhibited the expression of inflammatory molecule monocyte chemoattractant protein‐1 (MCP‐1) and fibrotic TGF‐β1 signaling, and eventually reduced the size of the infarcted area.^40^ In myocardial ischemia in mice triggered by isoproterenol administration, puerarin treatment greatly improved myocardial inflammation presentation, such as the reduction of infarcted area via an inhibition of the NF‐κB inflammatory downstream cascading molecules tissue necrosis factor alpha‐1 (TNF‐α1), interleukin 1B (IL‐1B), and interleukin‐6 (IL‐6), preventing the development of cardiomyocyte apoptosis.^41^

Interestingly, puerarin treatment decreases myocardial apoptosis, prevented arrythmia, and improved myocardial ischemia in diabetic rats, and further study shows that these therapeutic effects were closely associated with the increase of the eNOS expression, NO production, and antioxidant molecule SOD expression and the decrease of inflammatory TNF‐α1 expression simutaneously.^42^ In diabetic rats, puerarin also reduced the myocardial infarction area via a reduction of oxidized stress, an inhibition of NF‐κB inflammation, and a potential rise in vascular endothelial growth factor A (VEGFA) mediated angiogenesis, subsequently greatly improving cardiac performance.^42^ In addition, puerarin administration significantly improved cardiac diastolic function in diabetic cardiomyopathy, which was related to an increase of endogenous antioxidant enzyme activity and a reduction of the NF‐κB inflammatory signaling.^43^ From a clinical view, in a rat coronary arterial disease model, puerarin lowered the circulating levels of cardiac injury related molecules creatine kinase (CK), creatine kinase MB isoenzyme (CK‐MB), lactate dehydrogenase (LDH) and cardiac troponin, suggestion a reduction of cardiomyocyte necrosis or damage; and from a pathologic view, puerarin reduced the incidence of cardiomyocyte apoptosis via the activation of anti‐apoptotic BCL2/BAX pathway and the reduction of pro‐apoptotic caspase 3 activation.^44^ Taken together, these findings indicate that puerarin shows potential as a therapeutic molecule for the treatment of myocardial ischemia and myocardial infarction.

### 2.5 Cardiac hypertrophy and dysfunction

Heart failure is a complex syndrome that results from structural changes, such as cardiac arrythmia, cardiac hypertrophy, or functional impairment of ventricular function, which are markedly increased with advancing age.^5^ Angiotensin II (Ang II) treatment increased the serine/threonine protein kinase (AKT) and the mammalian target of rapamycin (mTOR) pathway, reduced an activation of autophagy, and importantly inhibited collagen secretion from atrial fibroblasts and collagen deposition, however, these adverse molecular and cellular effects were significantly alleviated or abolished by puerarin treament.^45^ Collagen aberrant deposition and aggregation in the atrial wall is the pathologic foundation of developing super‐ventricular arrythmia. Puerarin also retarded the development of ventricular hypertrophy and ventricular fibrosis through the upregulation of miR15b/195 and the suppression of the p38 MAP kinase/TGF‐β1 fibrotic signaling pathway.^46^ Actually, emerging evidence indicate that both miR15b and miR195 are novel anti cardiac hypertrophy and fibrosis molecules.^47^, ^48^ Interestingly, puerarin markedly increased expression of the anti‐oxidant nuclear factor erythroid factor 2 (NrF2) and restored hypertrophied cardiac function in hypertensive rats with an Ang II infusion.^49^ In myocardial fibrogenic mice induced by isoprenaline, a synthetic sympathomimetic amine, puerarin administration markedly inhibited the fibrotic TGF‐β signaling and inflammatory NF‐κB activation, blocked collagen deposition, and eventually substantially improved cardiac performance.^50^ Puerarin also significantly retarded the development of cardiac hypertrophy and fibrosis in ovariectomized rats of pressure overload via an upregulation of the PPARα/PPARϒ coactivator‐1α (PGC‐1) metabolic pathway.^51^ In addition, puerarin stabilized the sodium channel related action potential in hypertrophic human cardiomyocytes, which could potentially prevent the occurrence of cardiac arrythmias.^52^ These studies demonstrate that puerarin has therapeutic effects on cardiac arrythmia, cardiac hypertrophy, cardiac fibrosis, and cardiac dysfunction.

### 2.6 Brain aging, ischemic stroke, and cognitive decline

Aging is major risk factor for the initiation and progression of Alzheimer’s disease (AD); and the AD is the most common neurodegenerative disorder in the elderly.^53^ Ischemic stroke is the major risk factor for vascular‐related dementia in the aging population.^53^ The AD like mice treated with puerarin for 6 weeks displayed a significant improvement in learning memory, which may be associated with an increase of SOD and a decrease of ROS production in the hippocampus.^54^ It is well‐known that increased ROS produces cytotoxicity and facilitates a loss of neurons in the hippocampus. In mice with dementia induced by amyloid β 1‐42 (Aβ 1‐42) injection, puerarin treatment increased the expression of brain derived growth factor (BDGF), glutathione‐S‐transferase (GST), and SOD, whereas it decreased phosphorylated‐Tau and ROS production in the hippocampus and cerebral cortex, resulting in a decrease of shrinking nuclei, and swollen, eccentrically dispersed neural bodies, and an improvement of both learning and memory in mice.^55^ In addition, puerarin treatment promoted hippocampal neurogenesis and reduced neural loss in rats with dementia induced by D‐galactose; and puerarin treatment also significantly decreased Tau phosphorylation, and amyloid β aggregation, subsequently improving AD symptoms, such as learning and memory.^56^ In the amyloid precursor protein/presenilin 1 (APP/PS1) transgenic mice, puerarin increased NrF‐2 and its downstream antioxidant molecule heme oxygenase 1 (HO‐1) and alleviated oxidative stress damage, blocked amyloid beta (aβ) deposition, and rescued cognitive decline.^57^ Interestingly, puerarin effected on cholinergic enzyme, and increased choline acetyltransferase (ChAT) expression and activity in the hippocampus and dramatically decreased the deposition of amyloids in the brain in ovariectomized guinea pigs.^58^ More interestingly, in oligomer Aβ‐induced stress mice, puerarin effectively mitigated anxiety and prevented cognitive deficits.^59^

In local cerebral ischemic rats after administration of puerarin, this nature molecule was detected in the hippocampus, suggesting this herbal extract can successful pass the blood‐brain barrier and is a druggable target in the brain.^60^ In hypertensive rats treated with puerarin for 14 days, angiogenesis was significantly increased, blood perfusion was greatly improved, and impairment of pia mater was markedly prevented in the brain, greatly contributing to an enhanced capacity for learning and memory.^61^ In a rat vascular dementia model, induced by permanent occlusions of bilateral common carotid arteries, after 45 days of puerarin intraperitoneal injection, pathological observations demonstrated that hypoxia‐inducible factor 1 (HIF‐1), an index of ischemia, was markedly decreased while the learning and memory were significantly improved.^62^ In a rat model with middle cerebral arterial occlusion, puerarin administration markedly inhibited the AMPK/mTOR/uncoordinated 51‐like kinase (ULK1) autophagic pathway, reduced neuronal loss, and protected brain ischemia/perfusion injures.^63^ Puerarin improved the memory of rats with cognitive decline, which was closely associated with the attenuation of aβ deposition and apoptotic promoter Bad expression, contributing to the reduction of neuronal apoptosis and the enhancement of neurogenesis in the hippocampus.^64^, ^65^ Interestingly, puerarin administration also attenuated cerebral ischemic cognitive deficits with locomotor disorders in rats, and further study shows that these therapeutic effects were associated with an increase in the PI3K/ATK1/ glycogen synthase kinase (GSK‐3β) signaling pathway.^66^ Taken together, the above studies of mouse and rat models indicate that puerarin shows potential as a bioactive ingredient from food for the treatment of the age‐related brain disorders, such as brain ischemia, cognitive decline, and dementia.

## 3 PUERARIN MITIGATES CELLULAR INFLAMMATION

### 3.1 Endothelial cells

The endothelium is a critical interface between blood and tissues. Not surprisingly, ECs are pivotal to maintaining vascular homeostasis, and multiple vascular diseases arise from endothelial defect or dysfunction, including atherosclerosis and hypertension.^2^, ^4^ Ang II inhibited the proliferation and migration of endothelial precursor cells (EPCs), however, puerarin treatment blocked these harmful effects for endothelial repair.^67^ Further, puerarin increased telomerase activity, prevented telomere shortening, and eventually reduced the senescence of EPCs.^68^ Ang II reduced the proliferation and migration of ECs, accompanied by increases in the expression of senescence associated beta‐galactosidase (SA‐β‐gal) and inflammatory molecules intracellular adhesion molecule1 (ICAM‐1), vascular cellular adhesion molecule (VCAM‐1), TNF‐α1, IL‐6, and production of ROS while decrease in the expression of antioxidant factor NrF2, however, puerarin treatment completely blocked or alleviated these oxidative, inflammatory, and senescent effects.^69^ Additionally, puerarin inhibited phosphorylation of protein kinase B (PKB) and degradation of IκB kinase, blocked p‐38 MAP kinase and NF‐kB activation, reduced an uptake of lectin‐like oxidized low‐density lipoprotein (ox‐LDL; LOX‐1) and expression of IL‐8, and eventually increases the viability of ECs.^39^ Notably, puerarin has been reported to increase re‐endothelialization and significantly improve vascular function in a carotid arterial injury rat model, which was closely associated with the increase in the survival and number of circulating EPCs.^70^ Thus, the survival capability of both EPCs and ECs is the main determinant of maintaining the integrity of the endothelium.^71^

### 3.2 Vascular smooth muscle cells

Contraction and relaxation of the arterial wall is mediated by changes in VSMC behaviors. The phenotypic shift of VSMCs from a contractile to a synthetic state is a key cellular event for the development of arterial inflammation, thickening, and stiffening.^2^, ^4^ Puerarin inhibited the expression of PKC‐β2, Rac1, p47 phox, and p67phox subunits of NADPH oxidase and ROS production in VSMCs, and reduced the proliferation of VSMC induced by high dose of glucose in vitro; and importantly, puerarin markedly prevented VSMC cellularity in thickened neointima in the injured arterial walls in Zuker obese rats in vivo.^72^ Puerarin enhanced the viability of VSMCs treated with ox‐LDL, which was associated with an inhibition of the p‐p38, JNK, and NF‐kB stress inflammatory signaling pathway.^73^ Puerarin inhibited the proliferation of VSMCs induced by ox‐LDL via the blockade of ERK1/2 and p38 MAP kinase mitogenic signaling and the reduction of cell cycle driving protein PCNA expression.^37^, ^74^ It is known that an increase in the proliferation of VSMCs drives the arterial intimal medial thickening and the progression of atherosclerosis. Remarkably, puerarin has been reported to effectively block the osteochondrogenic transdifferentiation of VSMCs, and reduced the expression of the bone formation associated molecule master transcriptional factor runt‐related transcription factor 2 (RUNX2), non‐tissue specific alkaline phosphatase (ALP), and osteocalcin (OC).^75^ An increase in osteochondrogenic transdifferentiated VSMCs is the main cellular event for developing arterial calcification, which is an independent histochemical predictor of arterial stiffening.^76^

### 3.3 Macrophages

Monocyte‐derived macrophages with an overloaded cholesterol play a pivotal role in the initiation, progression, and destabilization of atherosclerotic plaques.^77^, ^78^ Circulating monocytes infiltrate into the arterial intimal and differentiate into macrophages. Both activated macrophages and inflammatory VSMCs accumulate and engulf ox‐LDL becoming lipid‐laden foam cells within the arterial wall, a cellular hallmark of atherosclerosis.^79^ Puerarin facilitated cholesterol efflux and decreased lipid accumulation in macrophages; and puerarin also upregulated the antioxidant thioredoxin system, inhibited ox‐LDL‐induced macrophage inflammation, lipid uptake, and foam cell formation.^80^, ^81^ These findings suggest that puerarin is an effective nature molecule to fight inflammatory foam cell formation.

### 3.4 Neurons

Maintaining enough functional neurons in the hippocampal region are the key to preserve normal cognition in the elderly. In a vascular dementia rat model induced by permanent occlusion of bilateral common arteries, puerarin treatment substantially prevented neuronal loss, which was linked to a decrease in ROS stress and anti‐apoptosis, and importantly this treatment markedly improved cognitive decline.^65^ In vitro study shows that an exposure of intracellular overloaded ROS neurons isolated from sporadic AD to puerarin markedly attenuated the apoptotic‐related caspase 3 activity, stress molecules p38, and JNK signaling, contributing to a decrease in neural damage.^82^ Consequently, puerarin administration markedly enhanced the survival capability of the overloaded ROS neurons while diminished the number of apoptotic neurons.^82^ Thus, puerarin is an intracellular ROS scavenger, suppressing oxidative stress, reducing apoptosis, and eventually increasing neuronal survival.

## 4 SUMMARY AND PERSPECTIVES

Growing evidence indicates that puerarin administration could alleviate or diminish the adverse cellular and extracellular structural and functional remodeling in the myocardium, brain, and arterial wall. Puerarin treatment could improve EC, cardiomyocyte, VSMC, and neuronal survival, inhibit VSMC proliferation, and prevent collagen secretion and deposition. These cellular and matrix beneficial effects markedly improves endothelial function, arterial stiffening, myocardial performance, and brain cognitive function through fighting oxidative stress and inflammation. These beneficial effects are the fundamental mechanism for retarding the clinical relevance of chronic inflammation in the heart, brain, arteries in the pathogenesis of obesity, type II diabetes, hypertension, atherosclerosis, myocardial ischemia, myocardial infarction, and cerebral ischemia, which are increased with aging (Figure 1). Importantly, meta‐analyses and systemic reviews of small randomized clinical trials in ischemic stroke and unstable angina pectoris indicate that puerarin is a promising herbal medicine for the prevention or treatment of cardiovascular and cerebrovascular disorders.^83^, ^84^ Thus, puerarin is a potent candidate molecule for combating adverse inflammatory events within the heart, brain, and arterial wall.

## CONFLICT OF INTEREST

All authors declared no conflicts of interest.

## AUTHOR CONTRIBUTIONS

L. Z. searched, reviewed, and wrote a draft. L.L. conceived and designed the topic or theme. M. W. conceived, designed, analyzed relevant literatures, and wrote the paper.
